# Sex-biased immunogenicity of a mucosal subunit vaccine against SARS-CoV-2 in mice

**DOI:** 10.3389/fimmu.2024.1386243

**Published:** 2024-05-21

**Authors:** Jianping Li, Kevin S. Hsu, Savannah E. Howe, Tanya Hoang, Zheng Xia, Jay A. Berzofsky, Yongjun Sui

**Affiliations:** Vaccine Branch, Center for Cancer Research, National Cancer Institute, National Institutes of Health (NIH), Bethesda, MD, United States

**Keywords:** SARS-CoV-2, mucosal vaccine, lung tissue-resident T cells, innate immunity, and sex differences

## Abstract

**Introduction:**

Current vaccines against COVID-19 administered via parenteral route have limited ability to induce mucosal immunity. There is a need for an effective mucosal vaccine to combat SARS-CoV-2 virus replication in the respiratory mucosa. Moreover, sex differences are known to affect systemic antibody responses against vaccines. However, their role in mucosal cellular responses against a vaccine remains unclear and is underappreciated.

**Methods:**

We evaluated the mucosal immunogenicity of a booster vaccine regimen that is recombinant protein-based and administered intranasally in mice to explore sex differences in mucosal humoral and cellular responses.

**Results:**

Our results showed that vaccinated mice elicited strong systemic antibody (Ab), nasal, and bronchiole alveolar lavage (BAL) IgA responses, and local T cell immune responses in the lung in a sex-biased manner irrespective of mouse genetic background. Monocytes, alveolar macrophages, and CD103+ resident dendritic cells (DCs) in the lungs are correlated with robust mucosal Ab and T cell responses induced by the mucosal vaccine.

**Discussion:**

Our findings provide novel insights into optimizing next-generation booster vaccines against SARS-CoV-2 by inducing spike-specific lung T cell responses, as well as optimizing mucosal immunity for other respiratory infections, and a rationale for considering sex differences in future vaccine research and vaccination practice.

## Introduction

Severe acute respiratory syndrome coronavirus 2 (SARS-CoV-2), the etiology of coronavirus disease 2019 (COVID-19), continues to pose devastating consequences for human health. It is estimated that there are more than 769 million confirmed cases of COVID-19 and over 6.99 million deaths to date worldwide. A total of more than 13 billion doses of vaccine have been administered to date to address this public health crisis. Those parenteral vaccines are developed in mRNA (1, 2), adenovirus-vectored (3), recombinant spike protein (4, 5), and inactivated virus-based vaccine platforms (6), and primarily focus on inducing virus-neutralizing antibodies, which are effective in protecting against COVID-19 disease (7). However, SARS-CoV-2 breakthrough infections continue to resurge because of waning immunity and poor cross-reactivity of antibodies to new virus strain variants leading to declining efficacy of first-generation vaccines against SARS-CoV-2 (8–10). Previous work suggests that T cells recognizing conserved viral proteins play a critical role in protecting against severe SARS-CoV-2 infection, particularly against emerging variants where neutralizing antibodies are less effective (11, 12). CD8+ T cells have been shown to induce protection from severe COVID-19 in cancer patients (13) and to control viral replication in breakthrough SARS-CoV-2 infection (14). Depletion of CD8+ T cells in the macaque model supports the protective role of CD8+ against SARS-CoV-2 challenge (15). Thus, the induction of durable T cell responses is an important layer in the development of next-generation vaccines.

Respiratory mucosal immunity is believed to be critical in protecting against SARS-CoV-2 viral infection in host airways. Spike-specific mucosal IgA responses are correlated with protection against Omicron breakthrough infection (16). Mucosal dimeric IgA induced by protein-based mucosal boost vaccination is correlated with protection against SARS-CoV-2 challenge in macaques (17, 18). Current parenteral vaccines, even the most prevailing messenger RNA vaccines, do not induce respiratory tract mucosal IgA and T cell responses (19). Viral vector-based mucosal booster vaccines, which are capable of inducing mucosal immunity when administrated locally, were approved for emergency use in China, India, and Russia (20). Viral vector vaccines are typically associated with safety concerns. Compared to extensive studies on systemic vaccines, developing a vaccine eliciting effective immunity locally in the upper and lower respiratory tracts remains underrepresented, particularly, a booster mucosal vaccine with fewer safety concerns such as a protein-based regimen. Developing a mucosal booster vaccine to prevent breakthrough infection due to escape from the antibody-mediated immunity warrants attention. Previous work in other respiratory virus infections suggests that lung resident memory T cells (T_RM_)are pivotal in protecting against influenza A virus and respiratory syncytial virus (20–22). CD8+ T_RM_ in respiratory tracts were also shown to mediate a protective role in controlling SARS-CoV-2 virus replication (14). Thus, it’s important to elicit long-term memory T cells in the mucosa, but it is tremendously challenging due to poor immunity in the respiratory mucosa (23).

Increasing evidence supports the idea that sex differences influence immune responses to SARS-CoV2 infection. Previous work has shown that there is a male bias for COVID-19 severity and mortality (24, 25). It is well known that sex differences affect immune responses to various types of vaccines, such as influenza vaccine, where females tend to elicit higher antibody levels than males (26). A clinical study showed that females administered with half a dose of influenza vaccine achieved stronger antibody responses than age-matched males who received full vaccine doses (27). Additionally, females develop more severe adverse events after immunization (AEFI) than males (28). These observations imply the necessity of considering sex differences in vaccine studies and vaccination practice. Sex differences in systemic IgG antibody responses to vaccination are overrepresented in the field of vaccinology. Sex-specific differences in cellular responses, particularly resident T cell immunity in the lung, have yet to be defined. Addressing this knowledge gap linking sex differences and cellular mucosal immunity in the lung may explain the phenomenon of males having more severe COVID-19.

In this study, we explored a mucosal booster vaccine platform administered intranasally. The platform comprises a protein-based vaccine formulated with a specific CP15 adjuvant combination, incorporating Toll-like receptor (TLR) agonists (CpG-ODN and Poly I: C) along with IL-15 in 1,2-dioleoyl-3-trimethylammonium-propane chloride salt (DOTAP) liposomes. Previous vaccine studies have demonstrated the effectiveness of this adjuvant combination in enhancing T cell responses and we have reported that CpG and poly I:C synergize because they signal through complementary transducers, MyD88 and TRIF, respectively (29–31). Our previous results have also shown this mucosal vaccine regimen containing CP15 adjuvant is effective in inducing mucosal dimer IgA protecting macaques and hamsters from SARS-CoV-2 infections (17, 18, 32, 33). The induction of T cell immunity locally in the lung by mucosal vaccines against SARS-CoV-2 is yet to be determined. Additionally, in a Syrian golden hamster model, we showed that females immunized with this vaccine platform showed better virus challenge outcomes (33). However, whether sex-biased immune responses, particularly T cell responses in the lung, were induced by the vaccine remains unclear. Herein, we found that mice intramuscularly primed with aluminum adjuvanted spike recombinant proteins and intranasally boosted with the mucosal booster vaccine elicited strong systemic antibody, nasal IgA, and BAL IgA responses, and most importantly robust T cell immune responses in the lung in a sex-biased manner that is independent of mouse genetic background. The data provide novel insights into optimizing next-generation booster vaccines against SARS-CoV-2 by inducing spike-specific tissue resident mucosal T cell responses, and a rationale for considering biological sex differences in future vaccine research and vaccination practice.

## Materials and methods

### Mouse immunization and sample preparation

Male and female C57BL/6 or BALB/c mice at 8 weeks of age were immunized with two doses of vaccines at day 0 and day 14. At day 0, all vaccine groups were intramuscularly primed with 10 μg SARS-CoV-2 S1 recombinant protein (Sino Biological, catalog # 40591-V08H) formulated with 10 μL aluminum phosphate gel (InvivoGen, catalog # vac-phos-250) in a 100 μL volume. The adjuvant control group was intramuscularly administered 100 μL aluminum phosphate gel only without S1 protein. PBS naive control received 100 μL PBS. At day 14, mice received vaccine boosts or control adjuvants by different routes of administration. For the intramuscular route of booster vaccine, mice were boosted with 10 μg SARS-CoV-2 S1 recombinant protein adjuvanted with either aluminum phosphate gel or a CP15 adjuvant. The CP15/S1 booster vaccine was prepared following the methodology outlined in a previous vaccine study (34). Initially, immunomodulators, including 10 µg of D-type CpG oligodeoxynucleotide (InvivoGen, catalog # vac-1826–1), 20 µg of Poly I: C (InvivoGen, catalog # tlrl-pic), and 5 µg of IL-15 (Peprotech, catalog # 210–15) protein, were dissolved in a desired volume of HBS (GIBCO) buffer within a microcentrifuge tube. In a separate tube, 10 µL of DOTAP (Roche, catalog # 11202375001) was added into the same volume of HBS. Subsequently, these solutions were mixed without agitation and incubated at room temperature for 20 minutes. Finally, the resulting CP15 mixture was combined with 10 µg of S1 prior to vaccination in the mice. For the mucosal route of booster vaccine, mice were intranasally administered 10 μg S1 protein mixed with a CP15 adjuvant in 40 μL. The Adjuvant control group was intranasally administered 40 μL CP15 adjuvant only. The PBS control group intranasally received 40 μL PBS. All animal studies were under the protocol VB-28 approved by the NCI IACUC.

At 2 weeks post-booster immunization, mice were humanely euthanized by inhalation of carbon dioxide in a chamber. Bronchoalveolar lavage (BAL) fluid was collected by flushing the lungs twice with 0.8 mL of PBS within a minute after euthanization. Nasal wash was collected by flushing the nasal cavity with 1 mL of cold PBS. Sera were obtained via cardiac bleeding. BAL and nasal fluids were centrifuged at 2000 rpm for 5 minutes, and the supernatants were harvested for assays.

### ELISA for the detection of S1-specific IgG and IgA antibody titer

ELISA plates (Greiner Bio-One, catalog # 675061) were coated with 100 ng/well SARS-CoV-2 spike S1 protein at 4°C overnight and followed by 3 washes with wash buffer (Seracare, catalog # 50–63-04). Plates were blocked with 3% BSA (Seracare, catalog # 50–61-10) for 1 hour at room temperature (RT). Serum samples were serially diluted 4x starting at 1:150 dilution. BAL (bronchoalveolar lavage) and nasal wash samples were undiluted. 50μL diluted sera or BAL or nasal wash samples were incubated with plates at RT for 1 hour. Plates were washed with 200 μL wash buffer 4 times. Goat anti-mouse IgG (Abcam, catalog # ab6789) and goat anti-mouse IgA (Abcam, catalog #97235) HRP-conjugated secondary antibodies were diluted 1:5000. 50 μL of diluted HRP-conjugate antibodies were applied into each well at RT for 1 hour. Plates were washed 4 times and air dried. 50 μL 3,3′,5,5′-tetramethylbenzidine (TMB) 2-component microwell peroxidase substrates (biotech-R&D systems, catalog # DY999) were added into each well for color development following the manufacturer’s instructions. Plates were developed at RT for 30 minutes and quenched with 50 μL 2N sulfuric acid stop solution (biotech-R&D systems, catalog # DY994). Absorbance was measured by a multi-mode microplate reader (Molecular Devices) at 450 and 550 nm. Endpoint titers for C57BL6 serum samples were represented by the area under the curve (AUC), while the reciprocal half-maximal inhibition concentration (IC50) was used to calculate antibody titers for BALB/c serum samples in Prism GraphPad software. Spike-specific BAL IgA could not be converted to ng/ml without a standard spike-specific IgA to create a standard curve. Total BAL IgA levels were quantified using an IgA mouse ELISA kit (Catalog # EMIGA), following the manufacturer’s instructions. In order to adjust for variability in total IgA isolated in BAL preparations, we normalized the spike-specific IgA to total IgA in each animal in **Supplementary Figure S1B**.

### Lung mononuclear cell isolation

Mouse lungs were perfused with perfusion buffer (0.5 M EDTA, 25 mM HEPES in PBS). Lung was finely cut up and digested with 1 mg/ml Collagenase D (Sigma, catalog # C5138–1G), 1 mg/ml Hyaluronidase (Sigma, catalog # H3506), and 50 U/ml DNase I (Sigma, catalog # D5025) in RPMI medium for 40 min at 37°C with shaking at 220 rpm. Tissues were mashed with a syringe plunger in a 100 μm cell strainer and filled up to 10 mL with PBS containing 10% fetal bovine serum (FBS). Tissue suspension was centrifuged for 5 min at 300xg and then the supernatant discarded. The pellet was resuspended in 10 mL 37% percoll (Sigma, catalog # P4937) and spun for 20 min at 2000rpm at RT with acceleration brake 5 and deceleration brake 0. The top layer and supernatant were removed. Red blood cells were lysed with ACK solution and followed by 2 washes at 400xg for 5 min with RMPI containing 10% FBS.

### Intracellular cytokine staining

SARS-CoV-2-specific T cells induced by vaccines were measured from freshly isolated lung mononuclear cells. Cells were stimulated with 2 μg/mL SARS−CoV−2 PepTivator Peptide Pools (Miltenyi Biotec, catalog # 130–126-700) in the presence of 0.15 μg/mL brefeldin A (BioLegend, catalog # 420601) at 37°C 5% CO_2_ overnight. The negative control was cultured without the S1 peptide pool and treated with the same quantity of brefeldin A. Positive assay control cells were treated with 10 ng/mL PMA (Sigma, catalog #P1585), 50 ng/mL ionomycin (Sigma, catalog # I0634) and 0.15 μg/mL brefeldin A. Cultures were harvested after *ex vivo* stimulation and washed with PBS for further surface and intracellular staining for flow cytometry analysis. Cells were first stained with blue viability dye (Thermo Fisher, catalog # L23105) and followed by cell surface staining with an antibody mixture, including CD3-PE-Cy5, CD4-BV650, CD69-FITC from BioLegend, and CD45.2-BUV563, CD103-BUV737 and CD8β-BUV805 from BD Biosciences. Then, cells were permeabilized and fixed using FOXP3/Transcription Factor Staining Buffer Set (Invitrogen TM, eBioscience, catalog # 00–5523-00), and followed by intracellular cytokine staining with IL-2-PE-Cy7, IFNγ-APC, TNFα-APC-Cy7 from BioLegend. Samples were run on BD FACSymphony A5 cytometer and results were analyzed with Flowjo according to the gating strategy described in **Supplementary Figure S2** (**Supplementary Figure S2**). Spike-specific T cell responses against SARS-CoV-2 in each animal were presented by the frequencies of cytokine-positive staining cells in each sample stimulated with the S1 peptide pool minus those in medium treatment control.

### Lung innate cell staining

Lung mononuclear cells were first stained with the blue viability dye and followed by cell surface staining for 30 min in ice with a panel of antibodies, including MerTk-BV605, CD64-BV421, Ly6C-APC-Cy7, I-A/I-E-PE, CD11c-PE-Cy5, Siglec-F-APC, and F4/80-Alexa 700 from Biolegend, CD103-BUV737, CD11b-BUV805, Ly6G-BV711, and CD45.2-BUV563 from BD Biosciences. Fixed samples were analyzed on FACSymphony A5 cytometer and results were analyzed with Flowjo. Innate cell populations were showed in the gating strategy described in **Supplementary Figure S3**.

### Statistical analysis

Statistical analysis was performed using GraphPad Prism 10.0.1. Non-parametric one-way ANOVA using the Kruskal-Wallis test, followed by corrected Dunn’s multiple comparisons tests, was applied to analyze experiments with more than 3 treatment groups. Comparison between two groups of treatments used the non-parametric Wilcoxon-Mann-Whitney test. Spearman was applied to analyze correlations between innate cell populations and mucosal Ab responses as well as T cell responses against the vaccine with a 95% confidence interval. All statistical tests were 2-tailed. A *p*-value ≤ 0.05 was considered statistically significant. The statistical significance was divided into four levels: *p* < 0.05 (*), *p* < 0.01(**), *p* < 0.001 (***), and *p* < 0.0001(****).

## Results

### Subunit vaccines administered locally and systemically induced robust spike-specific humoral responses against SARS-CoV-2

**Figure 1:**
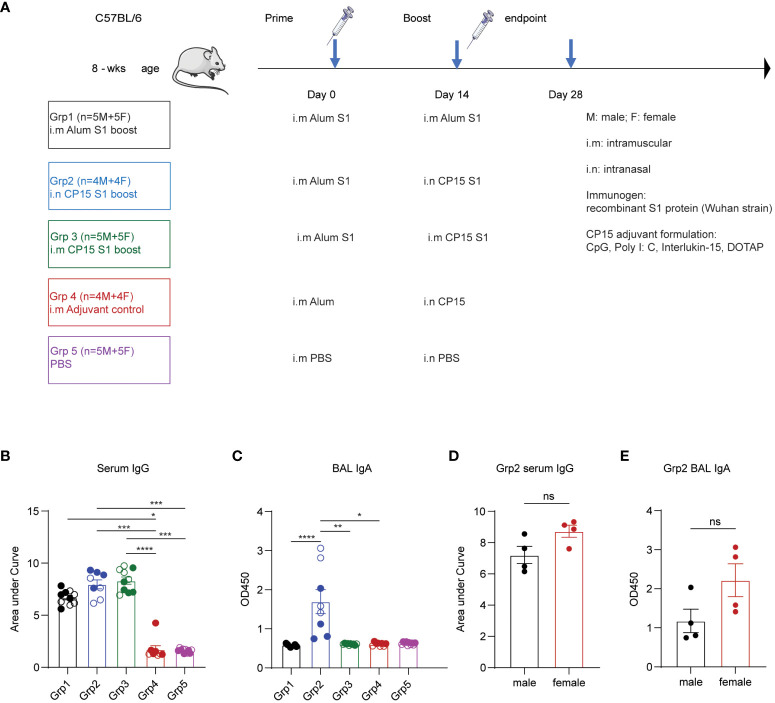
Vaccines induced spike-specific humoral responses in sera and bronchoalveolar lavage. **(A)** experimental timeline, treatment groups, animal numbers, and vaccination protocol. **(B)** IgG Ab titters in sera collected at two weeks post boost vaccination (day 28). **(C)** BAL IgA Ab responses at day 28 day. **(D)** comparison of male and female IgG responses. **(E)** BAL IgA Ab in males and females. Empty circle dots: female; Solid circle dots: male in **(B, C)**. Non-parametric one-way ANOVA using the Kruskal-Wallis test, followed by corrected Dunn’s multiple comparisons tests, was performed to analyze serum IgG and BAL IgA responses in group1–5 **(B, C)**. Data are representative of two independent experiments (n=4 or 5 per sex). The nonparametric Mann-Whitney test was used to compare sex differences in grp2 Ab responses **(D, E)**. The significance levels for p-values were denoted as follows: 0.1234 (ns), 0.0332 (*), 0.0021(**), 0.0002 (***), <0.0001 (****).

Five groups of mice with equal sex distribution were used to study the immunogenicity of vaccine regimens and vaccination strategies described in the experimental protocol (**Figure 1A**). A dose of 10 μg SARS-CoV-2 S1 recombinant protein was formulated in each vaccine regimen. All animals in vaccine groups (Groups 1–3) were primed with S1 protein emulsified in aluminum (alum) phosphate gel at day 0. The booster vaccines were administered systemically or locally on day 14: Grp 1 mice were intramuscularly (i.m.) injected with S1/alum, while grp 2 and grp 3 mice received S1/CP15- (CpG, Poly I: C, DOTAP, IL-15) intramuscularly (grp 3) or intranasally (grp 2). Group 4 was an intranasal CP15 adjuvant control group and Group 5 was a PBS control (i.m.) (**Figure 1A**). Two weeks after the vaccination, ELISA was performed to evaluate the production of spike-specific antibodies (Ab) including IgG in sera and IgA in BAL. The vaccine elicited significantly higher IgG Ab responses (**Figure 1B**). Spike-specific IgG responses in grp 2 and 3 were also higher than that of the alum-boosted grp 1, suggesting a better adjuvant effect of CP15 over alum in the booster injection regardless of route (**Figure 1B**). In BAL IgA responses, both spike-specific and total IgA levels in BAL samples were assessed via ELISA. The findings indicated no differences in total IgA concentration levels among groups, irrespective of vaccinations (**Supplementary Figure S1A**). Notably, elevated levels of spike-specific IgA Ab responses were only observed in animals from grp 2 (**Figure 1C**; **Supplementary Figure S1B**). The results were consistent with the previous studies that a systemic booster vaccine had a limited capacity to induce IgA responses. Females showed a trend of higher serum IgG (**Figure 1D**) and BAL IgA responses than did males (**Figure 1E**) in the grp2.

### The mucosal subunit vaccine elicited significant spike-specific cellular responses

**Figure 2:**
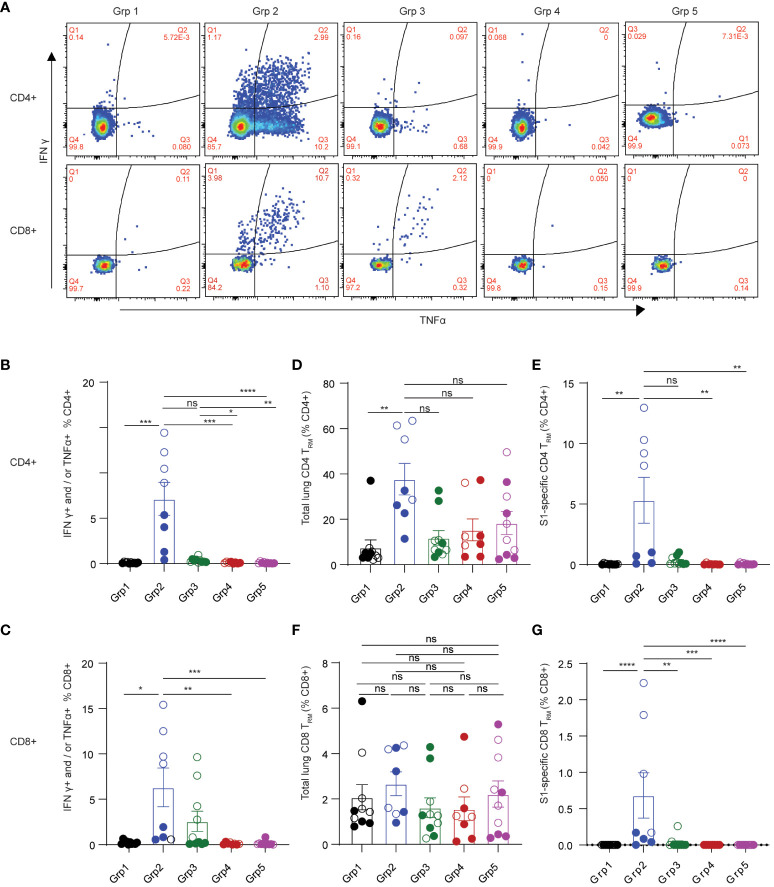
Spike-specific T cell responses in the lungs of C57BL/6 mice. **(A)** representative flow cytometry plots showing spike-specific T cell responses for each animal group. **(B)** spike-specific CD4+ T cell responses. **(C)** spike-specific lung CD8+ T_RM_. **(D, E)** total, and spike-specific lung CD4+ T_RM_, respectively. **(F, G)** total, and spike-specific lung CD8+ T_RM_, respectively. Empty circle dots: female; Solid circle dots: male in **(B–G)**. Data are representative of two independent experiments (n=4 or 5 per sex). Non-parametric one-way ANOVA using the Kruskal-Wallis test followed by corrected Dunn’s multiple comparisons tests was conducted in Prism to analyze cellular responses across groups 1–5. The significance levels for p-values were denoted as follows: 0.1234 (ns), 0.0332 (*), 0.0021(**), 0.0002 (***), <0.0001 (****) **(B–G)**.

Adaptive T cell responses against SARS-CoV-2 were reported as a key determinant of disease control (12). Therefore, induction of effective T cell immunity by vaccines in the respiratory tract is vital to combat SARS-CoV-2 infection at the site of invasion. We first evaluated spike-specific T cellular responses in the lung mononuclear cells stimulated with a SARS-CoV-2 S1 peptide pool by intracellular cytokine staining in the 5 treatment groups (**Figures 2A–G**). Spike-specific T cell responses were analyzed according to the gating strategy in **Supplementary Figure S2**. Only grp2 mice treated intranasally with the CP15 mucosal vaccine as a booster elicited significant spike-specific Th1 CD4+ responses (**Figure 2B**). The frequencies of antigen-specific IFNγ+ and/or TNF-α+ CD4+ T cells in the lung significantly increased in grp2 mice and reached 7.144% whereas the T cell response levels of grp1 (0.1%) and grp3 (0.35%) were on the same level as adjuvant and naïve control groups (grp4–0.1%, grp5–0.09%) (**Figure 2B**).

Interestingly, the CP15 adjuvanted intramuscular booster vaccine also elicited spike-specific CD8+ T cell responses in the lung (**Figure 2C**). However, CP15-adjuvanted mucosal booster vaccine (grp2) induced the strongest IFNγ+ and/or TNF-α+ CD8+ T cell responses in mouse lungs (6.3% in grp 2, **Figure 2C**), which were two-fold greater than those in grp3 with CP15 i.m. boost (2.58%) and almost 30-fold higher than in grp 1.

In contrast to the robust T cellular responses in the lungs (7.144% CD4+ and 6.3% CD8+ in grp 2), the T cell responses in the spleens were less potent. Spike-specific CD4+ and CD8+ T cell responses were 0.195% and 0.087%, respectively, for grp2, while there were no significant spike-specific T cell responses induced in the spleens of the other vaccine groups (**Supplementary Figures S1C, D**). These data highlighted that the mucosal booster vaccine not only effectively elicited strong mucosal T cell responses but also induced a modicum of systemic T cell responses.

Tissue-resident memory T cells are subsets of memory T_RM_, predominantly residing in barrier tissues (35). Pulmonary T_RM_ have been shown to limit the severity of SARS-CoV-1 infection (36, 37). Emerging evidence suggests that respiratory tract T_RM_ may contribute to protecting against SARS-CoV-2 infection (15, 38–40). Antigen-specific T_RM_ have been showed to be inducible by vaccination (37). Therefore, we evaluated the induction of pulmonary T_RM_ by the mucosal booster vaccine in mouse lungs. Based on the literature (35, 41–43), total or spike-specific lung CD4+ T_RM_ was defined by CD4+ CD69+ T cell expression with or without CD103, whereas pulmonary CD8+ T_RM_ was defined as expressing CD69 and CD103 (**Supplementary Figure S2** for gating strategies). Grp2 animals had a significant increase of both total and spike-specific lung CD4+ T_RM_ (**Figures 2D, E**). 37.705% of total lung CD4+ T_RM_ were detected in grp2, of which 5.317% of total lung CD4+ were spike-specific T_RM_. The frequencies of total lung CD4+ T_RM_ in grp 2 of intranasal vaccination were four-fold greater and three-fold greater than grp1 (7.581%) and grp3 (11.828%), respectively. Compared to a high level of lung spike-specific CD4+ T_RM_ in grp2 (5.317%), grp1 (0.036%) and grp3 (0.302%) showed marginal levels of responses. Although there were no significant differences in total lung CD8+ T_RM_ in all treatment groups (**Figure 2F**), grp2 had a significantly higher level of lung spike-specific CD8+ T_RM_ (0.683%) than grp1 (0%) and grp3 (0.0307%) (**Figure 2G**). Our results suggest that intranasal administration of CP15 adjuvanted protein vaccine induces strong T cell immunity locally in the lower respiratory tract in mice.

### Immunogenicity induced by the mucosal vaccine was sex-biased independent of mouse genetic background

**Figure 3:**
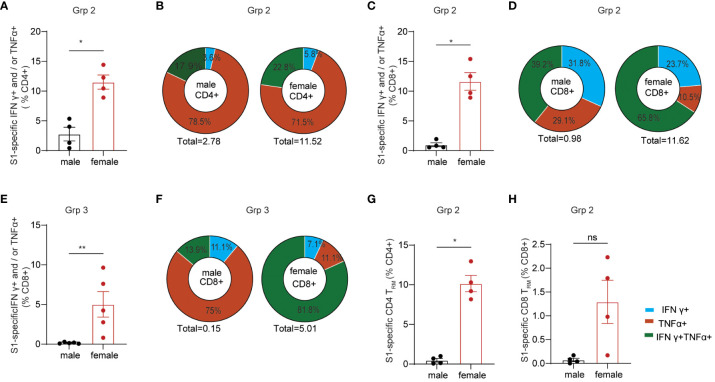
Sex-biased mucosal T cell responses induced by the protein mucosal vaccine in the lungs of C57BL/6 mice. **(A)** IFNγ+ and/or TNF-α+ secreting CD4+ T cell responses in male and female mice in i.n. boosted Grp2. **(B)** composition of spike-specific CD4+ T cells in i.n. boosted Grp2 of both sexes. **(C)** IFNγ+ and/or TNF-α+ secreting CD8+ T cell responses in i.n. boosted Grp2. **(D)** composition of spike-specific CD8+ T cells in i.n. boosted Grp2. **(E)** i.m. boosted Grp3 IFNγ+ and/or TNF-α+ secreting CD8+ T cell responses. **(F)** i.m. boosted Grp3 composition of spike-specific CD8+ T cells. **(G, H)** sex differences in spike-specific lung CD4+ and CD8+ T_RM_ in i.n. boosted grp2, respectively. Data are representative of two independent experiments (n=4 or 5 per sex). The nonparametric Mann-Whitney test was used to compare sex differences in T cell responses **(A, C, E, G, H)**. The significance levels for p-values were denoted as follows: 0.1234 (ns), 0.0332 (*), and 0.0021(**).

Females generally poduce more robust systemic IgG Ab responses than males after vaccination, which is observed in many types of vaccine regimens against a variety of viral and bacterial pathogens (27, 44). Sex-bias effects on cellular responses are less defined, especially in mucosal T cell responses induced by mucosal vaccination. Here, we primarily focused on determining the effects of sex differences on mucosal T cell responses in the grp2 vaccine platform as well as systemic Ab responses. Due to small animal numbers, sex-bias effects of Ab responses in vaccinated C57BL/6 mice were not observed in grp1 and 3 (**Supplementary Figures S1E–H**), but they showed a trend that females produced higher serum IgG and BAL IgA Ab responses than did males in the grp2 mucosal vaccine group (**Figures 1D, E**). We didn’t observe significant sex differences in splenic T cell responses against the spike in grp2 (**Supplementary Figures S1I, J**). Since only grp2 and grp3 treatment could induce mucosal spike-specific T cell responses, we further analyzed the sex effect on lung cellular responses in grp2 and grp3 mice. Our results showed that spike-specific T cell responses were more strongly induced in the lungs of females than males (**Figures 3A–H**). C57BL/6 female mice in grp 2 markedly elicited 11.51% and 11.63% IFNγ+ and/or TNF-α+ secreting CD4+ and CD8+T cells respectively, which is more than 4, and 11.86-fold higher than males (2.76%, and 0.98%) (**Figures 3A–D**).

We next investigated the polyfunctionality of the spike-specific mucosal T cell responses. Females had higher frequencies of polyfunctional T cells secreting both IFNγ and TNF-α as well as single IFNγ+ T cells than males did (22.75% vs 17.94% for S1-specific CD4, and 65.80% vs 39,17% for S1-specific CD8), while males had a higher frequency of single function TNF-α+ T cells (78.45%% vs 71.45% for S1-specific CD4, and 29.08% vs 10.53% for S1-specific CD8) (**Figures 3B, D**). Although no IFNγ+ and/or TNF-α+ secreting CD4+ T cell responses were detected in grp3 mice i.m. administered with CP15/S1 booster vaccine (**Figure 2B**), grp3 females induced more than 30-folder higher spike-specific CD8+ in the lungs than did males (5.01% vs 0.151%) (**Figure 3E**). Consistent with the results in polyfunctional CD8+ T cell responses in the lungs of grp2, female mice in grp3 induced higher frequencies of polyfunctional CD8+ that were double positive for IFNγ and TNF-α than did males (81.84% vs 13.89% IFNγ+TNF-α+of total S1 specific CD8+), whereas males had a predominant higher profile of TNF-α positive CD8+ specific to the spike protein than females (75% vs 11.06% TNF-α+of total S1 specific CD8+) (**Figure 3F**).

Additionally, females also showed significantly (or trend of) higher levels of lung CD4+ T_RM_ and CD8+ T_RM_ responses specific to spike than did males (10.15% vs 0.48% for CD4+T_RM_ and 1.29% vs < 0.07% for CD8+T_RM_) (**Figures 3G, H**) even though no sex-bias was found for total lung CD4+ T_RM_ and CD8+ T_RM_ in vaccinated grp 2 (**Supplementary Figures S1K, L**) and naïve control grp 5 (**Supplementary Figures S1Q, U**). Moreover, no significant sex differences were found in lung CD4+ or CD8+ T_RM_ regardless of antigen-specificity in grp3 that intramuscularly received the CP15 booster vaccine (**Supplementary Figures S1M–P**). The results suggest that primarily only the intranasal booster mucosal vaccine has a major impact on the induction of spike-specific CD4+ T_RM_ and CD8+ T_RM_ in the lungs.

**Figure 4:**
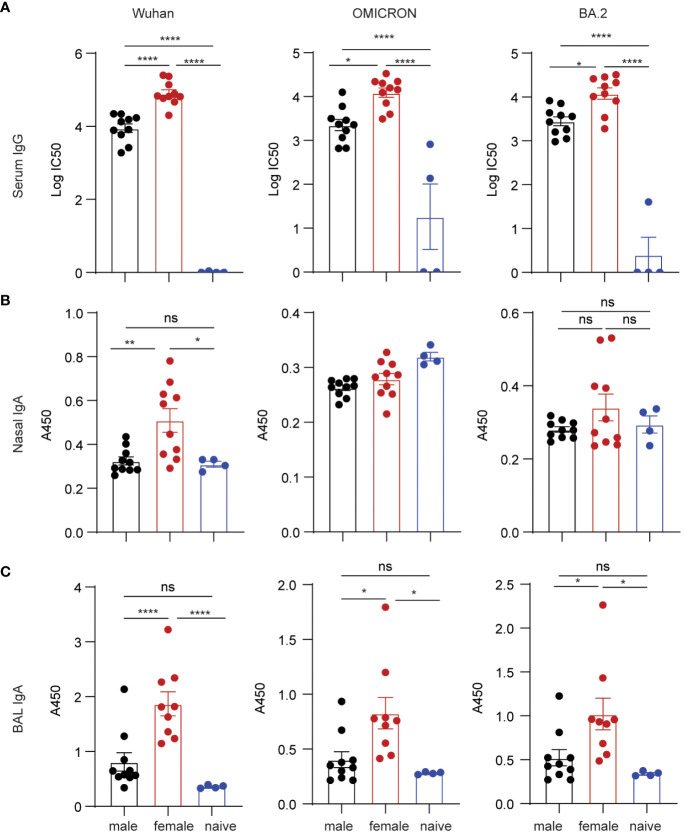
Spike-specific antibody responses in BALB/c mice vaccinated with mucosal boost vaccine. ELISA plates coated with S1 proteins included Wuhan, Omicron, and BA.2 strains. **(A)** IgG titers. **(B)** nasal wash IgA responses. **(C)** BAL IgA responses. Non-parametric one-way ANOVA using the Kruskal-Wallis test followed by corrected Dunn’s multiple comparisons tests was conducted in Prism to analyze cellular responses across vaccine and control groups **(A–C)**. Data are the pool of two independent experiments (n= 5 per sex). The significance levels for p-values were denoted as follows: 0.1234 (ns), 0.0332 (*), 0.0021(**), and <0.0001 (****).

It is well-known that C57BL/6 mice produce stronger Th1 immune responses, while BALB/c mice tend to produce stronger Th2 and antibody responses. To validate our results of sex-bias immunogenicity obtained in C57BL/6 and minimize the interference of genetic contributors, we further studied vaccine-inducing immunogenicity in BALB/c mice. Since only grp2 mice intranasally boosted with S1/CP15 mucosal vaccine generated strong mucosal responses, we tested only this vaccine platform in BALB/c mice. The experimental setup for this experiment paralleled that of experiments conducted in C57BL6 mice. The vaccine group of BALB/c mice underwent an initial i.m. systemic prime with Alum/S1 followed by an i.n. booster vaccine with CP15/S1, while the naïve control group received an i.m. prime with Alum only and an i.n. booster with CP15 only. Sera, nasal wash, and BAL samples from BALB/c mice immunized with the mucosal booster vaccine were collected two weeks after booster vaccination. ELISA was performed on those samples to examine spike-specific Ab responses against Wuhan, Omicron, and BA.2 virus strains. The mucosal booster vaccine elicited robust serum IgG responses against the Wuhan virus strain, Omicron, and BA.2 (**Figure 4A**; note the log scale). The IgG titers in females were almost 10-fold higher than those of males. For mucosal antibody responses, only female BALB/c mice showed significant nasal wash IgA Ab responses against the Wuhan strain but not against Omicron and BA.2. There were no detectable nasal wash IgA responses in male BALB/c mice compared to naïve animals (**Figure 4B**). Overall, females elicit significantly higher BAL IgA responses against the three tested virus strains than naïve control (Wuhan/p=0.005, Omicron/p=0.0229, BA.2/p=0.0323) but males did not (**Figure 4C**). The results showed that systemic IgG and mucosal IgA responses induced by the vaccine in BALB/c mice were sex-biased in females.

**Figure 5:**
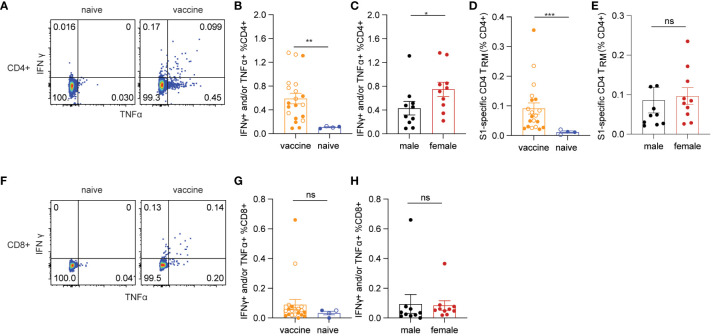
Spike-specific T cell responses in BALB/c mice. **(A)** representative flow cytometry plots of CD4+ T cell responses. **(B)** antigen-specific CD4+ T cells responses in vaccine and naïve groups. **(C)** sex differences in spike-specific CD4+ T cells responses. **(D)** spike-specific lung CD4+ T_RM_ in vaccine and naïve mice. **(E)** sex differences in spike-specific lung CD4+ T_RM_ in vaccinated mice. **(F)** representative flow cytometry plots of CD8+ T cell responses. **(G)** spike-specific CD8+ T cell responses in vaccine and naïve animals. **(H)** sex differences in spike-specific CD8+ T cells responses. The nonparametric Mann-Whitney test was used to compare the vaccine vs naïve group as well as sex differences in T cell responses **(B, C, D, E, G, H)**. Empty circle dots represent female mice whereas solid dots represent male mice in **(B, D, G)**. Data are the pool of two independent experiments (n= 5 per sex). The significance levels for p-values were denoted as follows: 0.1234 (ns), 0.0332 (*), 0.0021(**), and 0.0002 (***).

Next, we examined lung cellular responses in BALB/c mice vaccinated with the mucosal boost vaccine. In agreement with the data in C57BL/6 mice, the mucosal vaccine significantly induced spike-specific IFNγ+ and/or TNF-α+ CD4+ T cell responses compared to naïve control (p=0.0051) (**Figures 5A, B**). The frequencies of female spike-specific CD4+ T cells were almost twice that of their male counterparts (p=0.0288) (**Figure 5C**). Consistent with the results in C57BL/6 mice, spike-specific lung CD4+ T_RM_ were significantly induced by the mucosal booster vaccine (p=0.0002, **Figure 5D**), in which females showed a trend of a higher level CD4+ T_RM_ cell frequencies than males (p=0.0947, **Figure 5E**). The mucosal vaccine boost did not significantly elicit spike-specific IFNγ+ and/or TNF-α+ CD8+ T cell responses in vaccine groups and only two animals had positive responses (**Figures 5F–H**). Since there were no spike-specific CD8+ cellular responses induced in this study, we cannot evaluate the sex-biased effect on them here. Collectively, these results confirmed the female-biased effect on CD4+ T cell responses induced by the vaccine.

### Innate immunity was associated with the sex-biased immunogenicity of the mucosal vaccine

**Figure 6:**
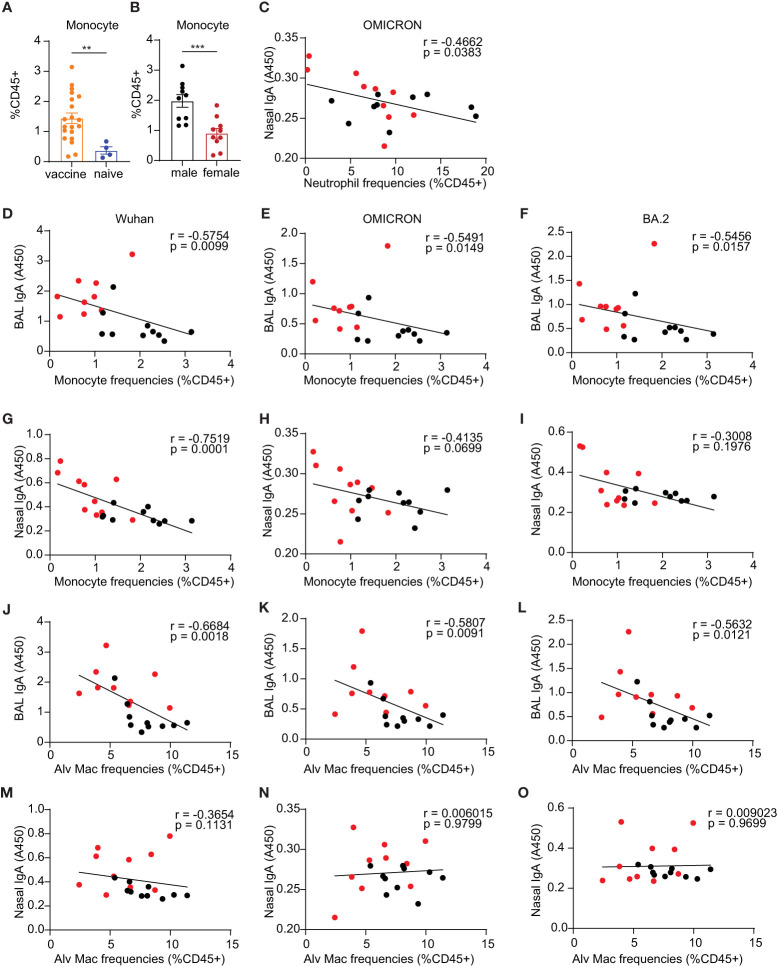
Innate immune responses of monocyte compartment in the lungs. **(A)** monocytes in the vaccine and naïve group. **(B)** comparison of the frequencies of monocytes in vaccinated males and females. **(C)** Spearman correlation between neutrophil frequencies and nasal IgA antibody responses against OMICRON strain. **(D–F, G–I)** Spearman correlation between monocyte frequencies and mucosal BAL and nasal Ab responses, respectively. **(J–L, M–O)** Spearman correlation between alveolar macrophage frequencies and mucosal BAL and nasal Ab responses, respectively. The nonparametric Mann-Whitney test was used to compare the vaccine vs naïve group and sex differences in monocyte responses **(A, B)**. Data are the pool of two independent experiments (n= 5 per sex). The significance levels for p-values were denoted as follows: 0.0021(**), and 0.0002 (***). Two-tailed Spearman was applied to analyze correlations between innate cell populations and mucosal Ab responses with a 95% confidence interval.

The innate immune system is the immediate defense line to alert the body to combat pathogen infection. It also plays a critical role in establishing effective and robust adaptive immune responses to vaccination. The mucosal vaccine platform in this study includes TLR agonists plus IL-15 as adjuvants, in which we demonstrated the adjuvant strategy can enhance trained innate immunity protecting against SIV transmission and SARS-CoV-2 viral challenges in the vaccinated macaque models in our previous studies (17, 18, 33). To determine whether the adjuvant combination would have a sex-biased effect on changing the frequencies of innate cells after vaccination and further influencing the adaptive immune responses to the vaccine, we analyzed the innate compartments in the lungs of both C57BL/6 and BALB/c mice administered the mucosal vaccine using gating strategies as described in **Supplementary Figure S3**. We observed a significant increase in F4/80+Ly6C int-hi monocytes in the vaccine group compared to naïve controls (p=0.0054) (**Figure 6A**) in BALB/c mice, while the levels of monocytes remained unchanged in the vaccine group compared to naïve controls in C57BL/6 mice (**Supplementary Figure S4E**). Neutrophils, a well-known innate population with negative impacts on CD4 T cells (45), exhibited a significantly reduced level in the vaccine group compared to the naïve control (p=0.03) in C57BL/6 mice (**Supplementary Figure S4F**), a trend not observed in BALB/c mice (**Supplementary Figure S4S**).

Interestingly, BALB/c males showed significantly greater frequencies of monocytes than females (p=0.0007) (**Figure 6B**), whereas C57BL/6 male and female mice had comparable frequencies of lung monocytes (**Supplementary Figure S4L**). C57BL/6 male mice demonstrated lower levels of natural killer cells (NK cells) compared to females (p= 0.03) (**Supplementary Figure S4N**), while there are no sex differences in NK cells in BALB/c mice (**Supplementary Figure S4Z**). The mucosal boost vaccination did not significantly change the frequencies of lung alveolar Macrophages (Alv Macs) (**Supplementary Figures S4A, O**), interstitial macrophages (Interstitial Mac) (**Supplementary Figures S4B, P**), DCs (**Supplementary Figures S4C, Q**), lung resident CD103+ DCs (CD103+ DCs) (**Supplementary Figures S4D, R**), and natural killer cells (NK) (**Supplementary Figures S4G, N**). Males showed a trend of slightly higher frequencies of Alv Mac (**Supplementary Figures S4H, U**) and neutrophils (**Supplementary Figures S4M, Y**) but lower DCs (**Supplementary Figures S4J, W**) and CD103+ DCs (**Supplementary Figures S4K, X**) than females.

To assess whether the alterations in innate immune cell frequencies correlated with adaptive immune responses, Spearman analysis was conducted. This analysis was exclusively carried out in BALB/c mice due to insufficient power to perform correlation analysis in the C57BL/6 experiments. Among these innate populations examined, we demonstrated significant correlations between monocytes and alveolar macrophages and mucosal antibody responses but not in NK cells, DCs, and CD103+ DCs Nasal wash IgA had a significantly inverse correlation with neutrophils for the Omicron strain (p=0.038, **Figure 6C**). The F4/80+Ly6C int-hi monocyte compartments in the lungs demonstrated significantly inverse correlations with mucosal antibody responses of BAL IgA against the three tested viral S1 proteins, including Wuhan (p=0.01, **Figure 6D**), Omicron (p=0.015, **Figure 6E**) and BA.2(p=0.016, **Figure 6F**). For nasal wash IgA, a significant correlation was also observed in antibody responses against the Wuhan strain with the monocyte compartment (p=0.0001, **Figure 6G**), but there were no significant correlations with Ab responses against OMICRON and BA.2 strains (**Figures 6H, I**). These results suggest that monocyte responses may play a role in diminishing the vaccine-induced antibody responses in males. Alv Macs were also shown to be inversely correlated with BAL IgA responses against virus strain Wuhan (p=0.002, **Figure 6J**), Omicron (p=0.009, **Figure 6K**), and BA.2 (p=0.012, **Figure 6L**). Nasal wash IgA was not correlated with Alv Macs (**Figures 6M–O**). These findings also suggest that Alv Macs may negatively affect the mucosal antibody production induced by the vaccine.

**Figure 7:**
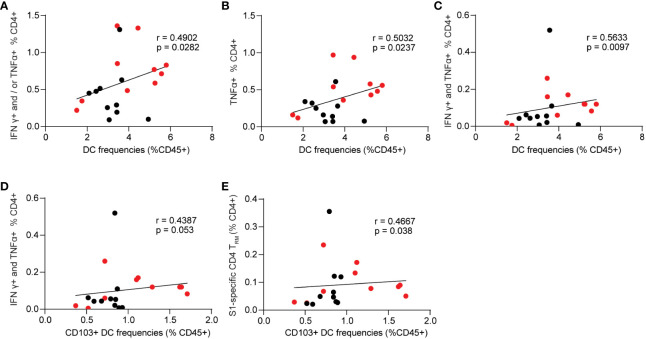
Spearman correlation between innate cell frequencies and spike-specific CD4+ T cell responses. **(A–C)** Dendritic cell frequencies and singular or polyfunctional S1-specific CD4+ T cells. **(D)** correlation between CD103+ dendritic cells and S1-specific CD4+ T cells that are double positive for IFNγ and TNF-α. **(E)** correlation between CD103+ dendritic cells and spike-specific lung CD4+ T_RM_. Data are the pool of two independent experiments (n= 5 per sex). Two-tailed Spearman analysis was applied to perform correlations between innate cell populations and mucosal Ab responses with a 95% confidence interval.

Next, we evaluated the correlations between innate cell frequencies and cellular responses in the BALB/c mice (**Figures 7A–E**). While monocytes and alveolar macrophages exhibited significant correlations with mucosal antibody responses, we did not observe similar significant correlations between these cell populations and S1-specific cellular responses. Among the innate populations examined, we only identified significant correlations between total lung DCs and CD103+ DCs with S1-specific cellular responses. The results showed that lung DCs were positively correlated with spike-specific CD4+ secreting IFNγ+ and or TNF-α+ (p=0.0282, **Figure 7A**). The frequencies of spike-specific TNF-α+ single functional and IFNγ+ TNF-α+ double positive CD4+ T cells have significant positive correlations with total lung DCs (p=0.0237, **Figure 7B**; p=0.0097, **Figure 7C**). Importantly, CD103+ resident DCs were also significantly correlated with spike-specific IFNγ+ TNF-α+ polyfunctional CD4+ T cell responses, and CD4+ T_RM_ in the lungs (p=0.053, **Figure 7D**; p=0.038, **Figure 7E**).

## Discussion

Currently approved COVID-19 vaccines are administered parenterally and induce a robust systemic immune response to protect against severe disease. Systemic routes of vaccination are generally poor inducers of mucosal immunity, which can only be induced following a mucosal (local) route of immunization (46). The majority of pathogens enter the body via a mucosal surface, meaning that the mucosal immune system provides the first line of defense to protect against infections. Mucosal immune responses are at least partly compartmentalized, and the route of vaccination (or infection) influences the localization of activated immune cells, although there is some crosstalk between sites (47). For example, a nasal route of infection would predominantly generate a response in the respiratory tract and salivary glands (48). This is due in part to immune cell “homing” of imprinted T and B lymphocytes induced in a particular inductive mucosal site, such as the NALT or GALT. Thus, an intranasal immunization route would be effective at generating a protective antibody (sIgA) and cellular (local resident memory T cells) response to protect against SARS-CoV-2 infection. This topic has been addressed in several thorough reviews in recent years (49–51). Despite this, it’s extremely challenging to induce mucosal immunity locally in respiratory mucosa. Antigens delivered to mucosa would be easily trapped by mucosal gel, diluted in mucosal secretions, degraded by protease and nuclease, and have limited ability to cross epithelial barriers, which would result in inefficient priming of the immune cells locally (23). Here, we used a CP15 adjuvanted mucosal booster, which has been shown to protect antigens from degradation and effectively elicit mucosal immunity in mice (29) and protected macaques and hamsters from SARS-CoV-2 challenges (17, 18, 33), to investigate whether it can induce sex-biased cellular responses in the lungs.

We found that mice intranasally administered with S1/CP15 mucosal booster vaccine elicited significant spike-specific CD4+ and CD8+ T_RM_ cell responses in the lung, in addition to mucosal IgA that recognizes SARS-CoV-2 ancestral and variant spike proteins in the lung and nasal washes compared to systemic booster vaccines. This is consistent with the common consensus in the field of vaccinology that parenteral vaccines are less likely to provoke mucosal T cell responses than mucosal-administered vaccines (20, 52). Previous reports demonstrated that lung T_RM_ conferred protection against SARS and MERS coronavirus infection (36, 37). Emerging works showed that respiratory tract CD4+ and CD8+ T_RM_ contributed to limiting the severity of COVID-19 (15, 38–40). We envisioned that the lung CD4+ and CD8+ T_RM_ specific to the spike induced by the mucosal booster vaccine might contribute to the high efficacy of protection observed in the macaque and hamster models (17, 18, 33). Overall, Mucosal vaccine regimens may be ideal candidates for optimizing T cell immunity with current booster vaccines, particularly mucosal T cell immunity in the lung that is pivotal in resolving SARS-CoV-2 infection in the lower respiratory tract.

Recent studies of sex differences in immune responses against vaccines have been heavily focused on systemic antibody responses, and it remains elusive at the respiratory mucosa if sex-biased immune responses, especially cellular immunity, exist. In this study, we found females had higher lung spike-specific T cell responses than their male counterparts. Our data demonstrated that female mice tended to have a higher proportion of spike-specific polyfunctional T cells as well as lung CD4+ T_RM_ than did males, implying that it is likely the reason why female hamsters showed a better challenge outcome in our previous hamster study (33). We further confirmed that females had stronger systemic antibody responses than males when primed intramuscularly with alum/S1 and intranasally boosted with CP15/S1. This observation is consistent with previous findings that female mice respond better to S1 protein vaccination than males (53). Interestingly, we also found that females intranasally boosted with the S1/CP15 mucosal vaccine induced much more robust mucosal antibody responses, including BAL IgA and nasal wash IgA against Wuhan, Omicron, and BA.2 virus variants than did males. Sex differences in innate immune compartments in the lung following mucosal vaccination were also observed. The S1/CP15 booster vaccine induced significantly higher frequencies of the lung monocyte population in BALB/c mice. The results are consistent with the previous reports that intradermal injection of CpG-formulated vaccines expands Ly6C-hi monocytes in draining lymph nodes (54, 55). In contrast to the fact that males had higher monocytes than females, females showed slightly higher frequencies of lung dendritic cells. Moreover, neutrophils, a well-known innate population with negative impacts on CD4 T cells (45), revealed a similar trend in C57BL/6 mice as the observation of monocytes in BALB/c mice. Future studies will be needed to determine whether the higher levels of inflammatory innate cells such as monocytes and neutrophils in males contribute to their diminished immune responses. The sex-biased immune responses against the mucosal vaccine regimen are independent of genetic background since experiments performed in both C57BL6 and BALB/c mice showed consistent results.

Spearman analysis revealed that Alv Macs, monocytes, and neutrophils are significantly inversely correlated with mucosal IgA responses (**Figure 6**). Specific lung T cell responses positively correlated with lung DCs as well as resident CD103+ DCs (**Figure 7**). This data suggested that innate immunity cells in the lung might affect the induction of mucosal humoral and cellular immunity. We found that S1/CP15 mucosal vaccine-induced innate responses, especially myeloid cells, were associated with sex-biased adaptive immune responses. Monocytes showed a significantly male-biased excess in our study. Previous evidence also showed high levels of inflammatory monocytes could suppress vaccine immunity (56), especially antibody responses against influenza (57). Considering that monocyte frequencies were inversely correlated with mucosal IgA responses, monocytes may play a role in dampening immunity induced by the mucosal vaccine in males and render sex differences in mucosal antibody responses. However, how sex influences monocyte-T cell crosstalk requires further investigation. In addition, CD103+ resident DCs are the primary antigen-presenting and sampling cells and migrate to airway-draining lymph nodes to prime adaptive responses. Vaccine adjuvants such as CpG, Poly I: C, and IL-15 are known to stimulate both innate and adaptive immune compartments to improve vaccine effectiveness (58–60). CpG and Poly I: C are well-studied to polarize a Th1 response. Toll-like receptor 9 (TLR9), a receptor of CpG, was reported to exhibit *TLR9* promoter polymorphisms (SNP) rs5743836 that were associated with allele-specific *TLR9* mRNA regulation by estrogens in women, which was associated with spontaneous clearance of HCV infection (61). IL-15 promotes memory T cell formation. Induction of lung CD4+T_RM_ requires appropriate local antigen presentation and IL-15 signaling in the lung parenchyma niche (35, 62). Whether these adjuvants preferentially activate CD103+ resident DCs to secrete IL-15 and differentially present local antigens to prime or boost CD4+ T cells in females via a specific mechanism remains unknown and warrants further investigation.

Overall, this study demonstrated that a potent S1/CP15 mucosal booster vaccine achieved robust potentially protective mucosal immunity in mice, particularly lung spike-specific T cell responses with a female bias. This work provides novel insights into optimizing next-generation booster vaccines against SARS-CoV-2 by adding a mechanism of inducing spike-specific lung T cell responses and focusing both antibody and T cell responses on the upper and lower respiratory mucosa where transmission and initial infection occur and provides a greater understanding of biological sex differences that should be taken into account in future vaccine research and vaccination practice.

## Data availability statement

The raw data supporting the conclusions of this article will be made available by the authors, without undue reservation.

## Ethics statement

The animal study was approved by National Cancer Institute IACUC. The study was conducted in accordance with the local legislation and institutional requirements.

## Author contributions

JL: Conceptualization, Data curation, Formal analysis, Investigation, Methodology, Project administration, Software, Supervision, Validation, Visualization, Writing – original draft, Writing – review & editing. KH: Methodology, Writing – original draft, Writing – review & editing. SH: Writing – review & editing, Data curation, Formal analysis, Methodology, Validation, Visualization, Writing – original draft. TH: Writing – original draft, Writing – review & editing. ZX: Writing – original draft, Writing – review & editing. JB: Conceptualization, Supervision, Writing – original draft, Writing – review & editing, Funding acquisition, Resources. YS: Conceptualization, Supervision, Writing – original draft, Writing – review & editing.

## Supplementary material

Supplementary Figure 1 Spike-specific T cell responses in the spleens and comparison of sex differences in lung resident memory T cell responses. **(A)** total concentrations of BAL samples in all treatment groups. (**B**) spike-specific IgA as normalized to the total BAL IgA in all groups (OD_450_/ng of total IgA). **(C, D)** splenic CD4+ and CD8+ T cells that are specific to the spike, respectively. **(E, F)** Grp1 serum IgG and BAL IgA responses, respectively. **(G, H)** Grp3 serum IgG and BAL IgA responses, respectively. **(I, J)** sex differences in splenic CD4+ and CD8+ T cells that are specific to spike, respectively. **(K, L)** sex differences in total lung CD4+ T_RM_ and CD8+ T_RM_ in grp2, respectively. **(M, N)** sex differences in total lung CD4+ T_RM_ and CD8+ T_RM_ in grp3, respectively. **(O, P)** Grp3 sex differences in total lung CD4+ T_RM_ and CD8+ T_RM_, respectively. **(Q, U)** Grp5 sex differences in total lung CD4+ T_RM_ and CD8+ T_RM_, respectively. Empty circle dots represent female mice whereas solid dots represent male mice in **(A–D)**. Data are representative of two independent experiments (n=4 or 5 per sex). Non-parametric one-way ANOVA using the Kruskal-Wallis test followed by corrected Dunn’s multiple comparisons tests was conducted in Prism to analyze cellular responses across groups 1–5 **(A–D)**. The nonparametric Mann-Whitney test was used to compare the sex differences in Ab responses and cellular responses **(E–U)**. The significance levels for p-values were denoted as follows: 0.1234 (ns), 0.0332 (*), 0.0021(**), and 0.0002 (***).

Supplementary Figure 2 Gating strategies for intracellular cytokine staining and lung resident memory T cells.

Supplementary Figure 3 Gating strategies for lung innate cells.

Supplementary Figure 4 Innate cell frequencies in mouse lungs. Comparison of innate cell frequencies between the vaccine and naïve group in C57BL/6 mice **(A–G)** and BAB/c mice **(O–T)**. Sex differences in innate cell frequencies in C57BL/6 mice **(H–N)** and BAB/c mice **(U–Z)**. The nonparametric Mann-Whitney test was used to compare the innate cellular responses in the vaccine vs naïve group and sex differences in innate responses **(A–Z)**. Data are representative of two independent experiments (n=4 or 5 per sex) in C57BL/6 mice. Data are the pool of two independent experiments (n= 5 per sex) in BALB/c mice. The significance levels for p-values were denoted as follows: 0.1234 (ns), and 0.0332 (*).
